# Pursuit eye movements in dyslexic children: evidence for an immaturity of brain oculomotor structures?

**DOI:** 10.16910/jemr.13.1.5

**Published:** 2020-05-25

**Authors:** Simona Caldani, Christophe-Loïc Gerard, Hugo Peyre, Maria Pia Bucci

**Affiliations:** CNRS-Université Paris Nanterre, France; EFEE-Centre d'exploration fonctionnelle de l’équilibre chez l’enfant, Robert Debré Hospital, Paris, France; Child and Adolescent Psychiatry Department, Robert Debré Hospital, Paris, France; Paris Diderot University, France

**Keywords:** dyslexia, eye tracking, pursuit, brain immaturity, visual attentional process

## Abstract

*Background*: Dyslexia is a disorder found in 5–10% of school-aged children. Several studies reported visual deficits and oculomotor abnormalities in dyslexic children. The objective of our study was to examine horizontal pursuit performance in dyslexic children, despite its poor involvement in reading. *Methods*: Eye movements were recorded by video-oculography in 92 children (46 dyslexic children, mean age: 9.77 ± 0.26 and 46 non dyslexic, IQ- and age-matched children). Both the number of catch-up saccades occurring during pursuit task and the gain of pursuit were measured. *Results*: Catch-up saccades were significantly more frequent in the dyslexic group than in the non-dyslexic group of children. Pursuit performance (in terms of the number of catch-up saccades and gain) significantly improved with increasing age in the non-dyslexic children group only. *Conclusions*: The atypical pursuit patterns observed in dyslexic children suggest a deficiency in the visual attentional processing and an immaturity of brain structures responsible for pursuit triggering. This finding needs to be validated by neuroimaging studies on dyslexia population.

## Introduction

Dyslexia is a disorder found in 5–10% of school-aged children (1). The origin of dyslexia is still not well known; it is a complex reading disorder involving genetic and environmental factors (2). The hypothesis of a phonological deficit in dyslexia has been shared by several authors (3, 4, 5) but other researchers suggested that the phonological theory is partially correct and that it cannot explain all deficiencies reported in dyslexia and other theories have been proposed such as auditory, visual perception, working memory, and attentional abnormalities (6, 7, 8, 9, 10). For instance, several studies reported visual deficits and oculomotor abnormalities in dyslexic children, supporting the hypothesis of deficiencies in the magnocellular system (9, 11) and of a cerebellar impairment (12, 13). However Kronbichler, Hutzler & Wimmer (14) and Hutzler, Kronbichler, Jacobs & Wimmer (15) did not support the hypothesis of deficiencies of the magnocellular system. Our group further explored oculomotor capabilities in dyslexic children and suggested a deficit in visual attentional processing in relationship with an immaturity of cortical structures responsible for saccade triggering (16, 17, 18). Note however that some of the cited authors have conducted more recent studies in which they reported the difficulties to know if visual deficits are causal or consequential of dyslexia (19). These authors reported deficits in visual search as well as in auditory processing in children that were poor readers, suggesting that causes of dyslexia could be multiple, interacting and probabilistic, rather than deterministic. Finally, the hypothesis of an impairment of the visual system in dyslexia is still under debate given that some recent researchers think that this deficiency could be a consequence and not the cause of dyslexia (20, 21, 22). Studies done in monkeys by recording middle temporal visual area (MT) and medial superior temporal area (MST) (23, 24) reported that the magnocellular system provides the major input to cortical motion-processing during pursuit. Recall that pursuit eye movements are used to pursue a slowly moving small object. Subjects match the velocity of the eyes to the velocity of the object in order to keep its images on the foveae and to ensure online processing of visual signals during object movement (25). Frequently, when the eyes are not able to keep up with the motion of the object, subjects make saccadic movements (catch- up saccades) in order to reduce or eliminate the positional error. Moreover, in the literature, it has been shown that an essential prerequisite for a correct pursuit is maintaining attention, since diverting attention impairs pursuit performance, particularly the gain (26). In a more recent study, Chen, Valsecchi & Gegenfurtner (27), in order to study the relationship between attention and pursuit performances, recorded visual potentials and showed that the attentional engagement started with the beginning of the pursuit movement.

However, the mechanisms controlling both catch-up saccades and pursuits are still poorly understood, although common structures seem to be involved in their triggering. For instance, motor or position error signals in the superior colliculus could be shared by the saccadic and pursuit systems (28) and cerebellar lesions, particularly of the oculomotor vermis affect performance of both saccades and pursuits (29). At the cortical level, there is anatomical evidence for connections between structures responsible for saccades and pursuits (30). Pursuit movements in dyslexia have received relatively little attention, most likely because of their poor involvement in reading; however, exploring pursuit performance in dyslexic children could give some insight into the cortical/subcortical impairment and visual attention capabilities in the dyslexic population. Interestingly, Callu et al. (31) suggested that phonological awareness and pursuits could share common cognitive components, probably important for later reading. Pursuit can be stimulated and recorded in different ways and this is, most likely, the consequence of different results reported in the dyslexic population. Pavlidis (32) was the first to report, in a small group of dyslexic children (12), more erratic eye movements than control children when following sequentially illuminated light sources.

Black, Collins, DeRoach & Zubrick, (33) reported that pursuit performance was similar between dyslexic (N=26) and non-dyslexic (N=34) children, but that about 25% of dyslexics showed more intrusive saccades during pursuit movements. In addition, Eden, Stein, Wood & Wood (11) found poor pursuit in dyslexic children (twenty-six), particularly when pursuing a target moving from left to right and Fukushima, Tanaka, Jeremy, Williams & Fukushima (34), reporting poor gain of pursuits in children with dyslexia (N=18), suggested deficits in the frontal cortex, particularly frontal eye fields and supplementary eye fields, in line with electrophysiological studies done on monkeys (35, 36, 37). Other studies did not find such differences concerning between dyslexic and non-dyslexic children, most likely due to different methods used to stimulate and record pursuit eye movements (38, 39, 40, 41). Indeed Brown et al.(38) analysed only the presence of predictive eye movements during pursuit and its performance were just only subjectively judged. More precisely in this study eye movements were recorded by using a limbal sensing device (with low sampling rate of 50 Hz) and the quality of pursuit was qualitatively evaluated by a "blind" observer into seven categories, ranging from good to poor. Stanley, Smith & Howell (39) used five light-emitting diodes (LEDs) flashed sequentially one at a time with onset of the next light following immediately upon offset of the preceding light, at speed of 500/1000 ms and used an eye tracker with one/two degrees of absolute positional accuracy. Olson, Kliegl & Davidson, (40) recorded pupil and corneal reflection positions by using a television image. Ygge, Lennerstrand, Rydberg, Wijecoon & Pettersson (41) also evaluated smooth pursuit eye movement’s performance only subjectively and subjects were invited to follow the investigator’s index finger.

Based on all these findings, the goal of the present study was to record pursuits with an eye tracker with a precision of about 0.25° in a large group of French dyslexic children, and to compare these results with those obtained from an IQ-, age-matched non-dyslexic group of children. 

We want to explore further pursuits performance in this population by measuring the gain and the number of catch up saccades. Our hypothesis was that by comparing dyslexic children with age-matched non-dyslexic children we could report deficits in visual attentional capabilities in dyslexia population maybe due immaturity of central structures responsible of pursuit triggering. Note, however, that this study cannot resolve the open question where visual deficits are the cause or the consequence of dyslexia.

## Methods

Forty-six dyslexic children from 7.5 to 13 years old (mean age: 9.77 ± 0.26 years) participated in the study. Dyslexic children were recruited from Robert Debré pediatric hospital, to which they had been referred for a complete evaluation of their dyslexia including an extensive examination of their neurological/psychological and phonological capabilities. For each child, we measured the time they required to read a text passage, assessed their general text comprehension, and evaluated their ability to read words and pseudo-words using the L2MA battery (42). This is the standard test in France. It was developed by the “Centre de Psychologie appliquée de Paris” and is used to detect dyslexic populations. Inclusion criteria were scored on the L2MA, and were: more than two standard deviations from the mean, a normal mean intelligence quotient (IQ, evaluated using the Wechsler Intelligence Scale for Children-Fourth Edition, WISC-IV), namely between 85 and 115, and normal visual acuity for both distance vision and near vision (both eyes ≥10/10). Note that, as reported by Capano, Minden, Chen, Schachar & Ickowicz (43) the association between dyslexia and ADHD is high given that about 26% of ADHD had a comorbid reading disability. More recently, McGrath and Stoodley (44) in a meta-analysis reported an overlap of gray matter between dyslexia and ADHD in the right caudate that is related to share cognitive activities for executive functions and/or procedural learning. Consequently in the present study any hyperactivity deficit was excluded using the ADHD Rating Scale-parental report (ADHD-RS) (45).

Note also that the selection of children with dyslexia follows the criteria of DMS V even if recently Miciak & Fletcher (46) suggested to use a Multi-Tiered Systems of Support model (MTSS) for diagnosis of dyslexia. However, this model needs to be tested in further studies.

Forty-six chronological age- matched non-dyslexic children (mean age: 9.81 ± 0.33 years old) were also examined. The inclusion criteria were as follows: no known neurological or psychiatric abnormalities, no history of reading difficulty, no visual impairment, or difficulty with near vision. The recruitment of TD children was based on voluntary participation; they were sons and daughters of hospital employees. Also measure of IQ was estimated in the control group using two subtests, one assessing their verbal capability (similarities test) and one assessing their logic capability reasoning (matrix reasoning test). The normal range for both tests is 10 ± 3 (WISC-IV, 47). All the healthy children we tested had normal verbal (11.45 ± 0.8) and logic (10.94 ± 0.5) capabilities. Clinical characteristics of the two groups of children are shown in Table 1.

Table 1: Clinical characteristics of children

**Table 1:** Median and interquartile range of classification accuracy based on the k-means clustering of transition matrices.

	Dyslexic children N = 46	Non Dyslexic children N = 46
Chronological age (yrs)	9.8 ± 0.3	9.8 ± 0.3
Reading age (yrs)	7.8 ± 0.2	9.8 ± 0.5
Verbal IQ	99.7± 1.1	
Verbal Sc		11.45 ± 0.8
Logic IQ	98.9 ± 0.9	
Logic Sc		10.94 ± 0.5

The reading age of all children was assessed using the ELFE test (Evaluation of Fluency Reading) http://www.cognisciences.com/, Grenoble), in which the child have to read aloud a text for duration of one minute. Children came from different social and ethnic backgrounds, thus representing the French population.

The investigation adhered to the principles of the Declaration of Helsinki and was approved by the Institutional Human Experimentation Committee of CPP Ile de France I (Hotel-Dieu Hospital).

### Pursuit task

The pursuit task requires participants to follow a slowly moving visual target. Stimuli were displayed on a 22’ computer monitor. A red circular target waveform, approximately 0.5° of visual angle, was used twice with the same velocity (15°/s). The target was initially placed in the central position (0°) and then moved horizontally to one side until it reached the ±20° location, where it reversed abruptly and moved to the opposite side. A total of nine trials were run and included in the analysis. Children were instructed to keep their eyes on the target, wherever it moved. After a short break, children had to perform the same test again (48).

### Eye movement recording

Eye movements were recorded by a non-invasive system using infrared camera to record horizontal and vertical eye position independently and simultaneously for each eye: the Mobile EyeBrain Tracker (SuriCog). The PC monitor was placed 60 cm with a screen of 22 inch, the resolution was 1920×1080 and the refresh rate was 60 Hz. This eye tracker is a medical device EC-marked for medical purposes. Recording frequency was set to 300 Hz. The mobile EBT is linear and the precision of the system is 0.25 deg. Calibration was done at the beginning of each eye movement recording. Calibration was done under binocular viewing. During the calibration procedure, children were asked to fixate a grid of 13 points (diameter 0.5 deg) mapping the screen. Point positions in degree in the horizontal/vertical plans were: 20.9°/12.2°;0°/12.2°; 20.9°/12.2°;-10.8°/6.2°;10.8°/6.2°;-20.9°/0°; 0°/0°; 20.9°/0°;-10.8°/-6.2°;10.8°/-6.2°; -20.9°/-12.2°; 0°/-12.2° and 20.9°/-12.2°. Each calibration point required a fixation of 250 ms to be validated. A polynomial function with five parameters was used to fit the calibration data and to determine the visual angles. There is no obstruction of the visual field with the recording system and the calibrated zone covers a visual angle of ±22 deg.

### Procedure

Children were seated on a chair in a dark room, in front of a flat screen displaying the target stimulating pursuit eye movements. The head of the child was held straight with a headrest; viewing was binocular.

### Data analysis

Calibration factors for each eye were determined from the eye positions during the calibration procedure (48). The software MeyeAnalysis (provided with the eyetracker) was used to extract pursuit eye movements from the data. Detection of saccades during pursuit was based on criteria of minimum amplitude (2°) and velocity (30°/s). Catch-up saccades were defined as saccades in the target direction that served to reduce position error and to bring the eye closer to the target. The number of catchup saccades was counted for each trial. Amplitude was measured for each catch-up saccade. Pursuit gain corresponds to the relationship between the speed of the eye and the speed of the target and it was obtained by dividing eye velocity by target velocity for each trial. If its value is around 1 it means that the correspondence between the target and the eye is perfect. Scores were then averaged across trials for each test (48). Statistical analysis was performed with the Statistica software. In order to compare group differences, we used a univariate one-way ANOVA in which the dependent variables were the number of saccades and the gain, and the independent variable was the group. In the case of significant effects post-hoc Fischer’s test (LSD) was performed. The effect of a factor was considered significant when the p-value was below 0.05. Correlations between gain and catchup were examined in the two groups of participants with Pearson correlation coefficients (significant p-value below 0.05). A linear regression model was used for eye movements’ parameters (catch-up saccades and gain value) with the predictor variable being the chronological age in order to investigate the influence of chronological age on eye movements’ performances (significant p-value below 0.05).

## Results

**Figure 1.**
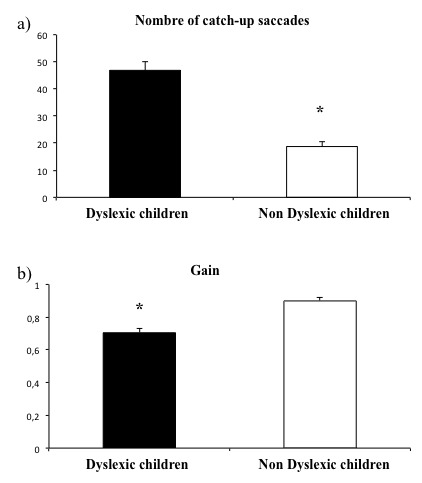
Mean of the number of catch-up saccades (A) and of the gain (B) measured during pursuits for dyslexic and non-dyslexic children examined Vertical bars indicate the standard error. * p < 0.05.

**Figure 2.**
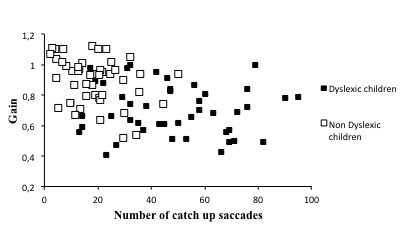
Gain value and the number of catch-up saccades measured for dyslexic and non-dyslexic children.

Figure 1 shows the mean number of saccades (A) and the gain (B) for both groups of children examined; ANOVA reported a significant group effect between the dyslexic and the non dyslexic children. The number of catch-up saccades occurring during pursuit movements was significantly higher in dyslexic children (F(1,90) = 58.26, p< 0.0001) and the gain of pursuits was significantly lower in dyslexic with respect to non-dyslexic children (F(1,90) = 35.2, p < 0.0001). Only for the non-dyslexic children a significant correlation between the number of saccades and gain was also found (R2= 0.09, p < 0.03) (see Figure 2).

**Figure 3.**
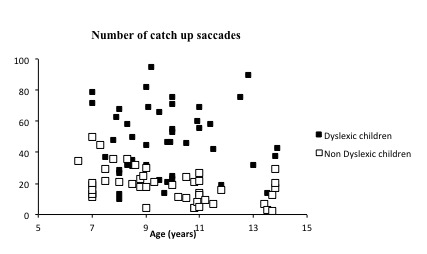
Number of catch-up saccades measured during pursuits for dyslexic and nondyslexic children examined.

Figure 3 shows the number of catch-up saccades measured during pursuits for dyslexic and non-dyslexic children examined as function of the age (in years) of each child tested. We found an association between number of saccades and age among children in the non-dyslexic group (R²=0.22 β = -2.27, p-value < 0.001), with a decrease of two saccades per year (in this group of children). However, we did not find any significant association for the dyslexic children (R2 = -0.000024, p = 0.9). The interaction of age x group was not significant for the number of saccades (F(2)=1.91, p = 0.1546).

**Figure 4.**
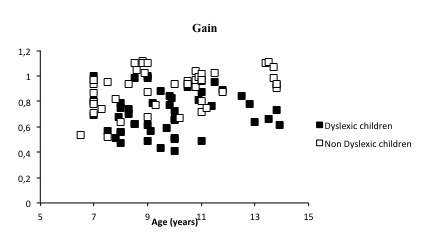
Gain (ratio of the eye velocity over the target velocity) for dyslexic and nondyslexic children examined.

Furthermore, the mean gain (ratio of the eye velocity over the target velocity, see Figure 4) increased significantly with the increasing age of the non-dyslexic children (R2=0.16, β =0.03, p<0.004).

 In other words, non-dyslexic children showed an improvement in the value of the gain of 3% every year. Concerning dyslexic children, however, the gain did not change with increasing the age (R2 = 0.008, p = 0.5).

We found also an interaction age x group for the value of the gain (F(2)=3.91, p < 0.0237), suggesting that the gain in non-dyslexic children improved with age compared to dyslexic children.

## Discussion

The main results reported in the present study were: i) dyslexic children showed a higher number of catch-up saccades and poor precision (that is lower gain) values during pursuit with respect to non-dyslexic age-matched children; moreover a significant correlation was found only in the group of non-dyslexic children; ii) Contrary to non-dyslexic age-matched children, dyslexic children did not show any improvement in pursuit performance with age. These findings are discussed below.

### Poor pursuit performance in dyslexic children

Dyslexic children, when compared to age-matched non-dyslexic children, showed a larger number of catch-up saccades and reduced gain values. This finding confirms and enlarges some previous studies done on a small number of children (11, 33, 34, 49). Nevertheless, some studies, probably because of the different methodologies used, found no differences between dyslexic and non-dyslexic children (38, 39, 40, 41). Research has suggested that an elevated number of catch-up saccades is observed when subjects have to compensate for pursuit deficiencies because the eye velocity is reduced, or increased, compared to target velocity (50). In the literature it has been shown that parietal areas are related to the suppression of saccades during pursuit as well as cerebellum (51). Moreover, microstimulation studies have shown that the oculomotor vermis could trigger saccades during pursuit (52, 53). Based on these findings, we could hypothesize that dyslexic children present dysfunctions in these cerebral areas, even if neuroimaging studies will be necessary to confirm our hypothesis. In line with that, the lack of correlation between the number of saccades and gain for the dyslexic children suggested the presence of an inhibitory control defect for this group of children. This finding could be explained by the presence of a deficit of prefrontal and fronto-striatal circuits that seem to be related to the ability to inhibit saccades during the pursuit task (25). Furthermore, reduced pursuit gain could be related to abnormalities in processing visual motion information in the extrastriate cortex, or in its visuo-motor transformation in the parietal or frontal cortex (54). In line with these studies, we could assume that dyslexic children present cerebral dysfunction also in these regions. Finally, as already mentioned in the Introduction section, attentional deficiencies could play a pivotal role in performing pursuit movements correctly. In the literature it has been reported that dyslexic children have a specific disability in orienting as well as in sustained focusing of visuo-spatial attention (7). In view of the fact that attention and eye movements share the same cerebral circuits as well as parietal and frontal areas (55) we could suggest an alteration of these cortical structures in dyslexic children. Moreover Biscaldi, Fischer & Hartnegg (56) showed that a specific visual attentional training in dyslexic children improved saccadic performance, particularly concerning voluntary saccade that are elicited by frontal lobe.

### Absence of improvement with age in pursuit performance in dyslexic children

In the literature it has been shown that the pursuit system is immature in the first years of life and that it improves until adolescence (57). These authors reported that the development of the cortical network correlated with eye movement triggering is in relationship with the processes of myelination, synaptic pruning and maturation of gray matter, which reaches adult levels in adolescence. Given the several cortical and sub-cortical structures implicated for pursuit triggering (58), we suggest that the absence of improvement of pursuit performances with age in dyslexic children could be due to immaturity of these areas responsible for pursuit realization. Moreover, as was mentioned in the introduction, we also have to point out that the attention system represents a cognitive factor that is active during pursuit, and in the literature it has been demonstrated that attentional functions are not mature yet in childhood. Indeed, Konrad et al. (2005), using fMRI, explored neural mechanisms of attention and showed that children, when compared to adults, reported significantly reduced neural activity in defined regions-of-interest for attentional mechanisms (particularly in right-sided frontal-midbrain regions during alerting, in the right-sided temporo-parietal junction during reorienting of attention, and in the dorsolateral prefrontal cortex during executive control of attention). It is also well known that dyslexic subjects show visuo-attentional difficulties (7), and impairment in attentional engagement has been observed under different tasks (59, 60, 61, 62). According to these studies, we could hypothesise that poor pursuit performance could be due to immaturity of cerebral areas controlling attention capabilities.

## Conclusion

Our data suggest that dyslexic children show atypical pursuit patterns (a lack of improvement in pursuit performance with age, a higher number of catch-up saccades and poor gain values) with respect to non-dyslexic age-matched children, probably due to an immaturity or dysfunction of brain structures responsible for pursuit triggering, as well as to a deficiency in the visual attentional processing.

## Limitations and directions for further research

In term of limitations we suggest that, in order to study and, eventually confirm, the presence of an interaction between age and group of children (dyslexic children as well as non-dyslexic age-matched children) in pursuit performance, it is necessary to replicate the same results on a larger sample. Moreover, in the future, in order to explore more deeply the presence of cerebral dysfunction and/or immaturity in dyslexic children we could use other oculomotor paradigms (as well as reflexive or voluntary saccade or fixation) that solicited others brain areas. Finally, it could be interesting to study the effects of a visual reading training on the cerebral area implicated in the triggering of pursuit; indeed, as shown by Olulade, Napoliello & Eden (63), there is a relationship between reading ability and the activity of area V5/MT during visual motion processing. Moreover in the future, it could be interesting to further explore the relationship between attentional capabilities and pursuit performances, given the high percentage of attentional deficits comorbidity in subjects with dyslexia (64).

## Ethics and Conflict of Interest

The author(s) declare(s) that the contents of the article are in agreement with the ethics described in http://biblio.unibe.ch/portale/elibrary/BOP/jemr/ethics.html and that there is no conflict of interest regarding the publication of this paper.
